# The hidden severity of new psychoactive substances: clinical evidence from emergency and intensive care admissions in Taiwan

**DOI:** 10.1016/j.aicoj.2026.100163

**Published:** 2026-09-28

**Authors:** Po-Hsiang Liao, Chih-Chuan Lin, Chia-Ming Chang, Sung-Wei Liu, Te-I Weng, Hsien-Yi Chen, Cheng-Chung Fang, Jiun-Hao Yu, Yu-Jang Su, Yen-Chia Chen

**Affiliations:** a Department of Emergency Medicine, Taipei Veterans General Hospital, Taiwan; b School of Medicine, National Yang Ming Chiao Tung University, Taipei, Taiwan; c Institute of Statistics, National Yang Ming Chiao Tung University, Hsinchu, Taiwan; d College of Medicine, Chang Gung University, Taoyuan, Taiwan; e Department of Emergency Medicine, Chang Gung Memorial Hospital, Linkou, Taiwan; f Department of Medical Education and Emergency Medicine, Hualien Tzu Chi Hospital, Buddhist Tzu Chi Medical Foundation, Hualien, Taiwan; g School of Medicine, Tzu Chi University, Hualien, Taiwan; h Department of Emergency Medicine, National Taiwan University College of Medicine and National Taiwan University Hospital, Taipei, Taiwan; i Forensic and Clinical Toxicology Center, College of Medicine and National Taiwan University Hospital, National Taiwan University, Taipei, Taiwan; j Institute of Forensic Medicine, College of Medicine, National Taiwan University, Taipei, Taiwan; k Department of Emergency Medicine, China Medical University Hsinchu Hospital, China Medical University, Hsinchu 30272, Taiwan; l Emergency Department, Mackay Memorial Hospital, Taipei, 10449, Taiwan; m Toxicology Division Emergency Department, MacKay Memorial Hospital, Taipei, 10449, Taiwan; n School of Medicine, College of Medicine, MacKay Medical University, New Taipei City, Taiwan; o MacKay Junior College of Medicine, Nursing, and Management, Taipei City, 11260, Taiwan; p Yuanpei University of Medical Technology, Hsinchu, Taiwan; q Department of Emergency Medicine, School of Medicine, National Defense Medical University, Taipei, 11490, Taiwan

**Keywords:** New psychoactive substances, Illicit drugs, Emergency department, Intensive care unit, Toxicology

## Abstract

**Background:**

New psychoactive substances (NPS) have emerged as a growing threat in Taiwan, yet their clinical severity compared with traditional illicit drugs (TID) remains uncertain. This study aimed to compare the clinical characteristics and critical outcomes between patients with NPS and TID poisoning confirmed by liquid chromatography–tandem mass spectrometry (LC–MS/MS).

**Methods and Results:**

We conducted a multicenter retrospective cohort study across 10 hospitals within six medical systems in Taiwan. Between March 2019 and January 2020, 1,378 patients with suspected drug intoxication underwent urine LC–MS/MS analysis at certified toxicology laboratories. A total of 362 patients (26.3%) tested positive, including 147 (40.6%) with NPSs, 130 (35.9%) with TIDs, and 85 (23.5%) with mixed substances.

Demographic, clinical, and laboratory data were collected through a chart review. Synthetic cathinones and ketamine analogs were the most frequently detected NPSs, whereas methamphetamine/amphetamine and morphine were predominant among TID cases. Critical outcomes were defined as intensive care unit admission or death in the emergency department. The overall rate of critical outcomes was 18.3% (252/1,378). The odds of critical illness did not significantly differ between NPS and TID users (adjusted odds ratio, 0.94; 95% confidence interval [CI], 0.51–1.74). Kaplan–Meier survival analysis revealed no significant difference in short-term survival among the NPS, TID, and mixed-drug groups (log-rank p = 0.665).

**Conclusions:**

No statistically significant difference in clinical severity or mortality risk was observed between NPS and TID poisoning. These findings highlight that NPS exposure has a similar potential for critical illness and should be managed with the same clinical vigilance as conventional drug poisoning.

## Background

New psychoactive substances (NPSs), a heterogeneous group of compounds, are designed to mimic the effects of classical illicit drugs while often evading legal control. Because of their synthetic nature and rapid chemical modification, NPSs are often undetectable using conventional urine drug screening assays [1]. The United Nations Office on Drugs and Crime (UNODC) defines NPSs as “substances of abuse, either in a pure form or a preparation, that are not controlled by the 1961 Single Convention on Narcotic Drugs or the 1971 Convention on Psychotropic Substances, but which may pose a public health threat” [2]. Globally, >130 countries have reported the emergence of one or more NPSs [2]. Ketamine occupies a distinct position within this framework. Although it has established medical uses and is frequently analyzed separately from other NPSs in clinical toxicology studies, it is not controlled under either of these international conventions and has been included within the UNODC NPS surveillance framework. In Taiwan, ketamine was initially the most prevalent NPS in the early 2000s; however, since 2017, synthetic cathinones have become the dominant category [3]. According to the UNODC Early Warning Advisory, NPSs comprise multiple structural groups, including aminoindanes, phencyclidine-type substances, phenethylamines, piperazines, plant-based compounds (khat, kratom, and *Salvia divinorum*), synthetic cannabinoids, synthetic cathinones, tryptamines, and other miscellaneous agents [1,2]. As these compounds are novel and not listed under traditional drug conventions, their toxidromes’ patterns are diverse. Clinically, reported cases of NPS poisoning encompass a wide spectrum of presentations, from mild agitation, hallucinations, and sympathetic stimulation to life-threatening complications [1,4,5]. Despite growing case reports and small series [[6], [7], [8]], systematic comparisons between NPSs and “traditional illicit drugs” (TIDs) in terms of risk of critical outcomes remain limited.

### Importance

Understanding whether NPS poisoning carries a lower, similar, or higher risk of critical outcomes, defined as intensive care unit (ICU) admission and emergency department (ED) death, compared with TID poisoning is of major clinical and public health significance. Although NPS-related presentations were relatively uncommon in some earlier ED cohorts [9], the toxicological profiles of NPSs are highly heterogeneous, and individual substances may cause severe or life-threatening toxicity. Certainly, users may consider NPSs less risky or more convenient than classical drugs, although their perceptions are inconsistent [10]. From a toxicological surveillance perspective, clarifying the comparative severity risk can inform emergency protocols, resource allocation, and legal/regulatory priorities. Moreover, given that many NPSs are newly emerging and regulatory control lags behind, establishing evidence of severe outcomes similar to TIDs would underscore the urgency of inclusion in screening panels and harm reduction strategies.

### Goal of this investigation

This study aimed to compare the clinical characteristics and outcomes of patients with NPS- and TID-related poisoning in multiple EDs in Taiwan. We analyzed the severity of poisoning, ICU admission rates, and survival outcomes to determine whether the risk of critical illness differs between NPS and traditional illicit drug exposures.

## Methods

### Study design, setting, and participants

The present study is a secondary analysis of a multicenter toxicological surveillance database previously used to characterize the clinical features of NPS exposure [5]. The previous analysis primarily compared NPS-positive with non-NPS drug-positive patients and excluded mixed NPS/non-NPS exposures from the primary comparison. The current study addresses a distinct clinical question focusing on critical outcomes and analyzes NPS-only, TID-only, and mixed exposures as separate groups.

This multicenter retrospective cohort study included patients who presented to the ED with suspected drug or substance poisoning across 10 hospitals within six medical systems in Taiwan. The participating institutions were MacKay Memorial Hospital (Taipei and Tamsui branches), Chang Gung Memorial Hospital (Linkou, Keelung, and Kaohsiung branches), National Taiwan University Hospital (Yunlin and Jinshan branches), Taipei Veterans General Hospital, Hualien Tzu Chi Hospital, and China Medical University Hospital. This study covered the period between March 2019 and January 2020 and was conducted in accordance with the Strengthening the Reporting of Observational Studies in Epidemiology statement.

### Data collection

Patients who were evaluated in the ED and underwent the NPS screening were included in this study. The indication for ordering an NPS test was based on the clinical presentation and physician judgment. Testing was performed in patients who exhibited abnormal levels of consciousness, demonstrated aggression or violence, attempted suicide, or presented with signs of sympathomimetic toxicity. Individuals with an unexplained altered mental status, a first episode of seizure without an identifiable cause, or a history suggestive of illicit drug use were also screened. In addition, patients involved in trauma with suspected substance use and those with out-of-hospital cardiac arrest aged <65 years were tested.

Patients with incomplete demographic information (age, sex) or unavailable final toxicological results were excluded. Patients who were legitimately prescribed controlled medications containing illicit components, such as morphine for cancer-related pain, were also excluded.

Urine samples were analyzed using liquid chromatography–tandem mass spectrometry (LC–MS/MS) at either the Clinical and Forensic Toxicology Center of National Taiwan University Hospital or Clinical Toxicology Laboratory of Taipei Veterans General Hospital. Exposure classification followed the UNODC-based framework used in the multicenter surveillance database [2], under which ketamine was included in the NPS category for the primary analysis. To address alternative classification conventions, an additional sensitivity analysis reclassified ketamine exposure, defined by detection of ketamine and/or its metabolite norketamine, as TID exposure, while novel ketamine analogues remained classified as NPS. The analytical panel did not include gamma-hydroxybutyrate (GHB). The benzodiazepines included within the NPS panel represented selected designer benzodiazepines rather than comprehensive screening for conventional benzodiazepines. Test ordering and patient selection were guided by emergency physicians’ clinical assessment and professional experience.

The demographic and clinical variables collected in this study included patient age, sex, and date of ED admission. The initial physiological parameters at triage included body temperature (°C), heart rate (beats per minute), respiratory rate (breaths per minute), systolic and diastolic blood pressure (mmHg), and peripheral oxygen saturation (%). Neurological status was assessed using the Glasgow Coma Scale (GCS), including eye opening, verbal and motor responses, and pupil size in both eyes. Medical comorbidities included chronic heart failure, chronic obstructive pulmonary disease, end-stage renal disease, and HIV infection. Behavioral and psychiatric manifestations were also documented. Presenting symptoms and chief complaints included first-time seizure, headache, palpitations, sweating, facial flushing, dyspnea, chest pain, nausea, or vomiting. Electrocardiographic (ECG) parameters included the PR interval, QRS complex duration, and QTc interval (ms). Laboratory test results included white blood cell count, platelet count, segment and lymphocyte percentages, hematocrit, blood urea nitrogen, creatinine, serum electrolytes (sodium, potassium, chloride), glucose, creatine kinase, liver function tests (glutamic-oxaloacetic transaminase, glutamic-pyruvic transaminase, total bilirubin), and arterial blood gas parameters (pH, partial pressure of carbon dioxide, and bicarbonate levels).

ED management variables included whether the patient was intubated, received inotropic agents, underwent cardioversion or hemodialysis, and underwent cardiopulmonary resuscitation. The patients’ ED disposition, length of hospital stay (days), and in-hospital mortality were also recorded as outcome measures.

### Statistical analyses

All statistical analyses were performed after a data quality assessment. Descriptive analyses were based on the available data for each variable, and no imputation was performed for missing values. Variables with substantial missing data (>20%) were excluded from the comparative analyses and reported descriptively. Arterial blood gas analysis and lactate, troponin, and creatine phosphokinase levels were selectively measured in patients with suspected severe poisoning, leading to higher missingness rates. For the remaining variables, distributional normality was examined using the Shapiro–Wilk test, which revealed that almost all continuous variables were skewed. Therefore, nonparametric tests were performed. Continuous data are summarized as medians with interquartile ranges (IQRs), whereas categorical variables are presented as counts and percentages.

Comparisons among the NPS, TID, and mixed groups were performed using the Kruskal–Wallis test for continuous variables and either the chi-squared or Fisher’s exact test for categorical variables, as appropriate.

Univariable and multivariable logistic regression analyses were performed to evaluate the association among NPSs, TIDs, and disease severity. The patients were classified into three exposure categories: NPS-positive only, TID-positive only, and mixed NPS + TID-positive. The outcome variable was critical illness, which was defined as requiring ICU admission or ED mortality. The criteria for ICU admission included severe acute organ dysfunction, inability to protect the airway, mechanical ventilation within the first 24 h, profound shock refractory to fluid resuscitation requiring vasopressors or inotropes, severe metabolic acidosis, and severe electrolyte disturbances [11]. Odds ratios (OR) and 95% confidence intervals (CI) were calculated to estimate the strength of association. A detectable-effect power analysis was performed to determine the odds-ratio magnitudes associated with 80% statistical power at a two-sided α level of 0.05 under the available sample size and model structure.

Sensitivity and subgroup analyses were performed to assess the robustness of the primary findings. First, because ketamine may be classified differently across toxicological frameworks, an alternative exposure classification was examined in which ketamine exposure, defined by detection of ketamine and/or its metabolite norketamine, was reclassified as TID, whereas novel ketamine analogues remained classified as NPS. Second, to account for potential between-center variation in ICU admission practices, the primary multivariable logistic regression model was additionally adjusted for participating medical center. The overall contribution of center was assessed by comparing the primary model with the center-adjusted model using a likelihood-ratio test. Third, because the sex distribution differed across exposure groups, potential effect modification by sex was evaluated by adding exposure-by-sex interaction terms to the age-adjusted logistic regression model. A global interaction test was performed, and sex-stratified logistic regression analyses were additionally conducted to estimate the association between exposure category and critical outcomes separately in male and female patients.

For the co-detection analysis, all unordered pairs of distinct substances detected within each patient were enumerated. Thus, a patient with *k* distinct detected substances contributed *k*(*k*-1)/2 pairwise co-detection events. Each substance pair was subsequently mapped to its corresponding drug category, and pairwise events belonging to the same category combination were aggregated. For example, concurrent detection of two different synthetic cathinones contributed to the cathinone–cathinone category, whereas concurrent detection of a synthetic cathinone and ketamine contributed to the cathinone–ketamine category. The frequency of each category-level pair was calculated relative to the total number of pairwise co-detection events. These aggregated frequencies were visualized using a chord diagram, in which each arc represented a drug category and each connecting ribbon represented a category-level co-detection pair; ribbon width was proportional to the number of corresponding pairwise co-detection events.

Finally, the survival outcomes were analyzed in patients with complete discharge data. Kaplan–Meier survival curves were constructed to compare survival probabilities between groups, and the log-rank test was used to assess statistical significance.

All statistical tests were two-tailed, and a p value <0.05 was considered statistically significant. The analyses were performed using Python (version 3.10; Python Software Foundation, Beaverton, OR, USA) with the Pandas, NumPy, SciPy, Lifelines, and Statsmodels packages.

## Results

A total of 1,384 patients underwent urine toxicology screening for suspected poisoning across the participating medical centers between March 2019 and January 2020. Six patients were excluded owing to missing ED disposition data (Fig. 1). Therefore, 1,378 patients were included in the final analysis. Of these, 252 (18.3%) were categorized into the critical outcome group, defined as those who required ICU admission (n = 245) and died in the ED (n = 7). The remaining 1,126 (81.7%) patients were classified into the noncritical outcome group, including those admitted to the general ward (n = 665) and discharged or transferred after ED management (n = 461). None of the patients was transferred to ICUs during hospitalization.

**Fig. 1:**
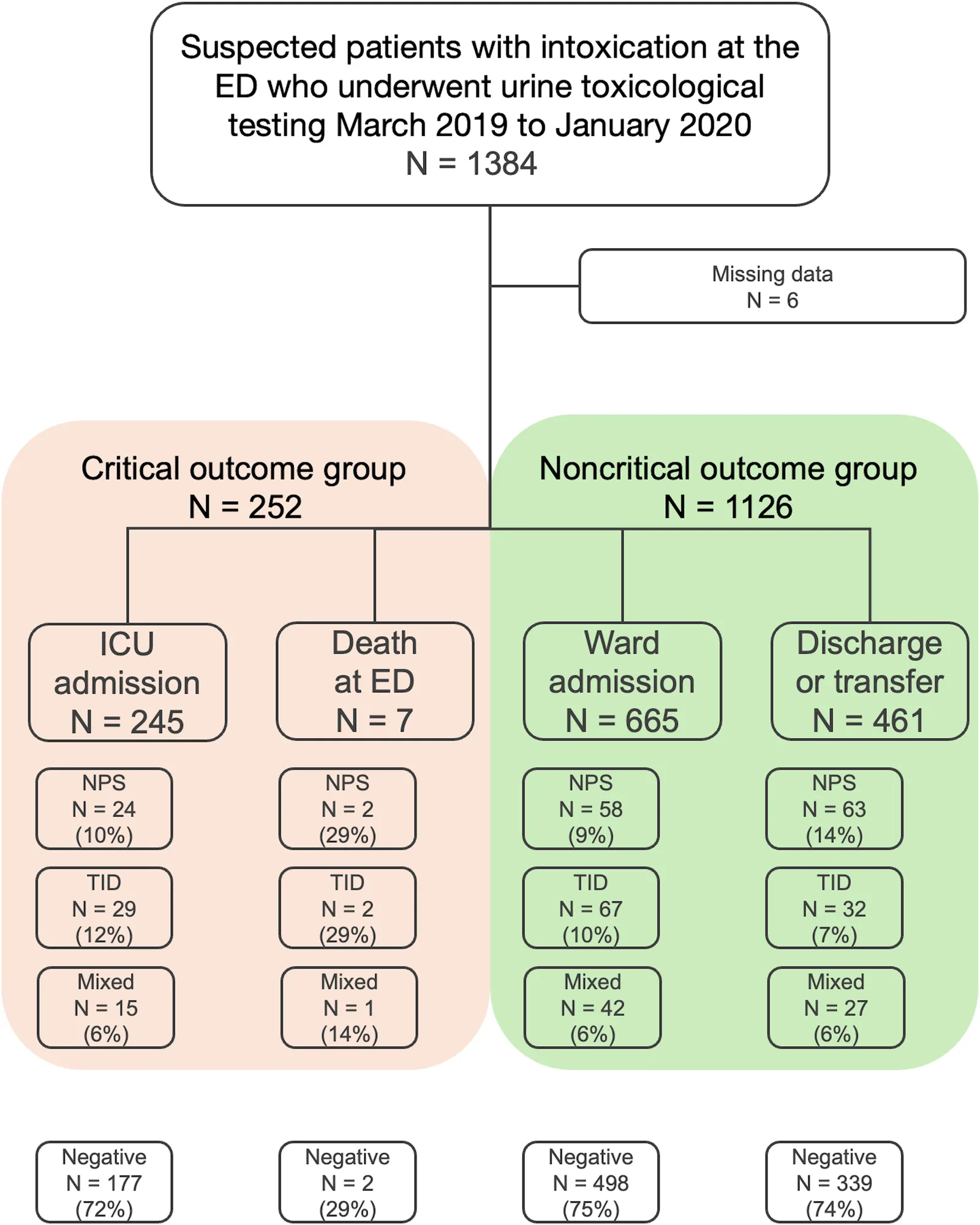
Flow diagram of patient enrollment and classification according to clinical outcomes. A total of 1,384 patients with suspected intoxication underwent urine toxicology screening in the emergency department (ED) between March 2019 and January 2020. Six patients were excluded owing to missing ED disposition data. The 362 patients with analytically confirmed NPS and/or TID exposure constituted the exposure-positive cohort used for comparisons among exposure categories. The red-shaded area represents the critical outcome group, defined as patients admitted to the intensive care unit (ICU) or those who died in the ED. The green-shaded area represents the noncritical outcome group, including patients admitted to general wards and discharged or transferred to another facility (For interpretation of the references to colour in this figure legend, the reader is referred to the web version of this article). ED, emergency department; ICU, intensive care unit; NPSs, new psychoactive substances; TIDs, traditional illicit drugs.

Among the 245 patients admitted to the ICU, 24 (10%) patients tested positive for NPS, 29 (12%) for TID, and 15 (6%) for both (mixed positive), whereas 177 (72%) had negative toxicology results. In the noncritical outcome group, 58 (9%) were NPS-positive, 67 (10%) were TID-positive, 42 (6%) had mixed results, and 498 (75%) were negative in the ward-admitted subgroup. Among the discharged or transferred patients, 63 (14%) were NPS-positive, 32 (7%) were TID-positive, 27 (6%) were mixed, and 339 (74%) were negative. The proportions of toxicology test positivity were 28.9% in the critical outcome group and 25.7% in the noncritical outcome group (p = 0.32), indicating no statistically significant difference in the proportion of positive urine toxicology results between the two groups.

Overall, 362 of the 1,378 patients (26.3%) tested positive for NPS and/or TID and constituted the exposure-positive comparative cohort, including 147 NPS-only, 130 TID-only, and 85 mixed NPS + TID exposures. Table 1 summarizes the baseline characteristics, clinical features, and outcomes of the patients with NPS, TID, and mixed substance exposure. A significant difference in sex distribution was observed across the three groups (p < 0.001); males predominated in the TID group (80.0%), whereas the NPS and mixed groups showed a higher proportion of females (44.9% and 37.6%, respectively). The patients in the NPS group were significantly younger than those in the TID group (median, 32 [24–45] vs. 43 [33–52] years; p < 0.001). The prevalence of HIV infection was markedly higher in the TID (10.0%) and mixed (9.4%) groups compared with the NPS (1.4%) group (p = 0.002).

**Table 1:** Baseline characteristics, clinical features, and outcomes of patients using new psychoactive substances (NPSs), traditional illicit drugs (TIDs), and mixed substance use (MIX).

	NPS (N = 147)	TID (N = 130)	MIX (N = 85)	P value
Demographics				
Sex				
Male	81 (55.10)	104 (80.00)	53 (62.35)	<0.001
Female	66 (44.90)	26 (20.00)	32 (37.65)	
Age (years)	32 (24–45)	43 (33–52)	33 (26–40)	<0.001
Congestive heart failure	5 (3.40)	3 (2.31)	0 (0.00)	0.279
COPD	3 (2.04)	2 (1.54)	0 (0.00)	0.460
ESRD	1 (0.68)	3 (2.31)	1 (1.18)	0.636
Liver cirrhosis	3 (2.04)	0 (0.00)	2 (2.35)	0.211
HIV infection	2 (1.36)	13 (10.00)	8 (9.41)	0.002
Physical examinations				
GCS score	14.0 (10.0–15.0)	14.0 (11.0–15.0)	15.0 (11.0–15.0)	0.248
Pupil size (mm)	3.0 (2.0–3.0)	3.0 (2.0–3.0)	3.0 (3.0–3.0)	0.580
Body temperature (°C)				
≥37.5 or <36	53 (36.05)	45 (34.62)	29 (34.12)	0.947
36–37.4	94 (63.95)	85 (65.38)	56 (65.88)	
Heart rate (/minute)				
>120 or <60	44 (29.93)	29 (22.31)	29 (34.12)	0.141
60–120	103 (70.07)	101 (77.69)	56 (65.88)	
Respiratory rate (/minute)				
>24 or <10	10 (6.80)	8 (6.15)	6 (7.06)	0.961
10–24	137 (93.20)	122 (93.85)	79 (92.94)	
Systolic blood pressure (mmHg)				
<90	9 (6.12)	13 (10.00)	5 (5.88)	0.386
≥90	138 (93.88)	117 (90.00)	80 (94.12)	
Peripheral oxygen saturation (%)				
<90	5 (3.40)	12 (9.23)	4 (4.71)	0.127
≥90	142 (96.60)	118 (90.77)	81 (95.29)	
Laboratory findings				
Creatinine (mg/dL)	1.0 (0.8–1.2)	1.0 (0.8–1.4)	1.0 (0.9–1.5)	0.660
Sodium (mmol/L)	139.0 (136.3–141.0)	138.0 (135.0–140.0)	139.0 (137.0–141.0)	0.114
WBC (×10⁹/L)	9.9 (7.1–13.3)	9.8 (7.4–12.6)	10.7 (7.6–16.0)	0.344
Platelet (×10³/μL)	276.0 (221.8–328.3)	252.5 (187.5–316.5)	285.0 (226.0–352.0)	0.037
Hct (%)	40.4 (37.0–44.8)	41.3 (36.3–44.2)	42.3 (38.0–45.5)	0.508
ECG PR interval (ms)	142 (126–160)	148 (134–164)	142 (129–156)	0.179
ECG QRS interval (ms)	86 (81–92)	88 (82–96)	88 (80–96)	0.393
ECG QTc (ms)	454 (430–483)	452 (433–474)	454 (433–471)	0.927
Clinical features				
Altered mental status	80 (54.42)	65 (50.00)	47 (55.29)	0.681
Bizarre behavior	35 (23.81)	29 (22.31)	24 (28.24)	0.602
Suicidal behavior	33 (22.45)	14 (10.77)	10 (11.76)	0.015
Self-harm	22 (14.97)	14 (10.77)	18 (21.18)	0.112
Violence	16 (10.88)	9 (6.92)	10 (11.76)	0.407
Delirium	38 (25.85)	28 (21.54)	27 (31.76)	0.244
Hallucination	12 (8.16)	10 (7.69)	5 (5.88)	0.810
First-time seizure	6 (4.08)	3 (2.31)	3 (3.53)	0.751
Status epilepticus	0 (0.00)	2 (1.54)	1 (1.18)	0.341
Myoclonus/rigidity	8 (5.44)	2 (1.54)	3 (3.53)	0.220
Sympathomimetic symptoms	13 (8.84)	13 (10.00)	7 (8.24)	0.898
Diaphoresis/sweating	11 (7.48)	4 (3.08)	10 (11.76)	0.040
Headache	7 (4.76)	5 (3.85)	1 (1.18)	0.370
Palpitation	11 (7.48)	2 (1.54)	6 (7.06)	0.038
Facial flush	8 (5.44)	4 (3.08)	6 (7.06)	0.373
Dyspnea	12 (8.16)	15 (11.54)	10 (11.76)	0.564
Chest pain	9 (6.12)	5 (3.85)	2 (2.35)	0.438
Nausea or vomiting	12 (8.16)	9 (6.92)	2 (2.35)	0.187
Tremor	5 (3.40)	7 (5.38)	6 (7.06)	0.450
Numbness	4 (2.72)	2 (1.54)	3 (3.53)	0.574
Focal weakness	1 (0.68)	4 (3.08)	0 (0.00)	0.175
Trauma case	8 (5.44)	8 (6.15)	8 (9.41)	0.485
Interventions				
Intubation at ED	19 (12.93)	18 (13.85)	9 (10.59)	0.778
Inotropic agent at ED	9 (6.12)	7 (5.38)	5 (5.88)	0.966
CPR	2 (1.36)	2 (1.54)	1 (1.18)	1.000
Cardioversion	1 (0.68)	1 (0.77)	0 (0.00)	0.594
Hemodialysis	1 (0.68)	0 (0.00)	0 (0.00)	1.000
ECMO	1 (0.68)	0 (0.00)	0 (0.00)	1.000
Clinical scoring				
APACHE II score	7.0 (3.0–12.0)	7.0 (4.0–14.0)	7.0 (4.0–11.0)	0.668
PSS score	1.0 (1.0–2.0)	1.0 (1.0–2.0)	1.0 (0.0–2.0)	0.834
0 (asymptomatic)	36 (24.49)	31 (23.85)	27 (31.76)	0.220
1 (mild)	48 (32.65)	53 (40.77)	22 (25.88)	
2 (moderate)	37 (25.17)	20 (15.38)	21 (24.71)	
3 (severe)	22 (14.97)	18 (13.85)	12 (14.12)	
4 (death)	4 (2.72)	8 (6.15)	3 (3.53)	
qSOFA score	1.0 (0.0–1.0)	1.0 (0.0–2.0)	1.0 (0.0–3.0)	0.929
Outcome				
Hospital stay (days)	3.0 (2.0–11.0)	8.0 (2.0–13.8)	4.0 (2.0–8.0)	0.044
ICU stay (days)	5.0 (4.0–8.3)	5.0 (3.0–11.0)	4.0 (3.0–14.5)	0.944
Mortality (%)	2.7%	6.2%	3.5%	0.392

NPSs, new psychoactive substances; TIDs, traditional illicit drugs; COPD, chronic obstructive pulmonary disease; ESRD, end-stage renal disease; GCS, Glasgow Coma Scale; ECG, electrocardiogram; Hct, hematocrit; WBC, white blood cell count; PSS, Poisoning Severity Score; APACHE II, Acute Physiology and Chronic Health Evaluation II; qSOFA, quick Sequential Organ Failure Assessment; CPR, cardiopulmonary resuscitation; ECMO, extracorporeal membrane oxygenation; bpm, beats per minute; mmHg, millimeters of mercury; ms, milliseconds; μL, microliter; mmol/L, millimoles per liter; mg/dL, milligrams per deciliter; HIV, human immunodeficiency virus.

Most vital signs and laboratory parameters, including GCS score, blood pressure, heart rate, and oxygen saturation, did not significantly differ among the groups. Platelet counts differed significantly across the three groups (p = 0.037), with the TID group showing a numerically lower median platelet count than the NPS group (252.5 × 10³/μL vs. 276.0 × 10³/μL). There were no significant differences in ECG parameters (PR, QRS, or QTc intervals).

Regarding clinical manifestations, suicidal behavior was more frequent among NPS users (22.5%) than among TID (10.8%) or mixed users (11.8%) (p = 0.015). Other neuropsychiatric features, such as bizarre behavior, hallucinations, and delirium, were comparable across the groups. Autonomic hyperactivity–related findings, including diaphoresis/sweating (p = 0.040) and palpitations (p = 0.038), differed significantly across the three groups.

Psychiatric and behavioral manifestations varied across NPS classes (Supplementary Table S1). Altered mental status was frequently observed among patients with synthetic cannabinoids (4/5, 80.0%), designer benzodiazepines (5/7, 71.4%), phenethylamines (20/32, 62.5%), and piperazines (41/68, 60.3%). Hallucinations and bizarre behavior were also observed across multiple NPS classes. However, several NPS subgroups were small, and these findings should therefore be interpreted descriptively.

No significant differences were observed in Acute Physiology and Chronic Health Evaluation II, Poisoning Severity Score, or quick Sequential Organ Failure Assessment scores among the three exposure groups. The median hospital stay was shortest in the NPS group (3 days [IQR, 2–11]) and longest in the TID group (8 days [IQR, 2–13.8], p = 0.044). The mortality rate was higher in the TID group (6.2%) than in the NPS (2.7%) and mixed exposure (3.5%) groups, but the difference was not statistically significant (p = 0.392).

Among all analytically confirmed cases, NPSs were identified in 232 patients, whereas TIDs were identified in 215 patients (Table 2). At the individual-analyte level, a total of 449 NPS detections were recorded. Within the NPS group, synthetic cathinones were the most frequently detected subclass (232 detections, 52% of NPS detections), followed by ketamine and its analogs (100 detections, 22%), piperazines (68 detections, 15%), phenethylamines (34 detections, 8%), designer benzodiazepines (8 detections, 2%), synthetic cannabinoids (5 detections, 1%), and tryptamines (2 detections, 0.4%). Among 416 TID detections, the most prevalent substances were methamphetamine (127 detections, 31%), amphetamine (101 detections, 24%), morphine (82 detections, 20%), and codeine (45 detections, 11%). Other commonly detected TIDs included 6-acetylmorphine, 3,4-methylenedioxymethamphetamine (MDMA), and cocaine metabolites. Notably, synthetic cathinones and ketamine analogs together comprised >70% of all NPS-related detections, whereas amphetamines and opioids were the dominant contributors in TIDs.

**Table 2:** Distribution of detected substances among patients using new psychoactive substances (NPSs) and traditional illicit drugs (TIDs) according to clinical outcomes.

Substance/drugs	Critical outcome (N)	Noncritical outcome (N)	Total (N)
New psychoactive substances (NPSs)^a^	42	190	232
Synthetic cathinones^a^	20	87	107
Eutylone	9	36	45
Mephedrone	7	30	37
Ephylone	5	21	26
Dibutylone	5	14	19
Methcathinone	2	15	17
Methylone	2	12	14
4-Chloromethcathinone	2	10	12
Ethylone	3	8	11
4-Chloro-N,N-dimethylcathinone	2	9	11
Methedrone	2	8	10
4-Methyl-α-ethylaminopentiophenone	3	6	9
4-Chloroethcathinone	1	5	6
Pentylone	1	0	1
3,4-Methylenedioxy-α-pyrrolidinobutyrophenone	0	4	4
Benzedrone	1	0	1
Butylone	1	1	2
4-Chloro-α-pyrrolidinovalerophenone	0	1	1
4'-Methyl-α-pyrrolidinohexiophenone	0	1	1
3,4-Methylenedioxy-N-tert-butylcathinone	0	1	1
3,4-Methylenedioxypyrovalerone	0	1	1
4-Ethylmethcathinone	0	1	1
Methylpentedrone	0	1	1
α-Pyrrolidinovalerophenone	0	1	1
Ketamine and analogues^a^	22	75	97
Ketamine and/or norketamine	22	75	90
Deschloroketamine	0	9	9
Deschloro-N-ethyl-ketamine	0	1	1
Piperazines^a^	10	58	68
mCPP	10	58	68
Phenethylamines^a^	4	28	32
N,N-DMA	2	22	24
MMA	1	6	7
6-Methoxy methylone	1	0	1
25B-NBOMe/ 2C-B-NBOMe	0	1	1
MDDMA	0	1	1
Designer benzodiazepines^a^	0	7	7
Phenazepam	0	6	6
Etizolam	0	2	2
Synthetic cannabinoids^a^	2	3	5
AMB-FUBINACA	2	2	4
AB-PINACA	0	1	1
Tryptamines^a^	0	2	2
5-MeO-DMT	0	1	1
5-MeO-MiPT	0	1	1
Traditional illicit drugs (TIDs)^a^	47	168	215
Methamphetamine	23	104	127
Amphetamine	19	82	101
Morphine	26	56	82
Codeine	11	34	45
6-Acetylmorphine	5	12	17
MDMA	5	17	22
Cocaine metabolite	3	8	11
MDA	3	6	9
THCCOOH	0	2	2

NPSs, new psychoactive substances; TIDs, traditional illicit drugs; mCPP, meta-chlorophenylpiperazine; N,N-DMA, N,N-dimethylamphetamine; MMA, 3-methoxy-4-methylamphetamine; 25B-NBOMe, 4-bromo-2,5-dimethoxy-N-(2-methoxybenzyl)phenethylamine; MDDMA, 3,4-methylenedioxy-N,N-dimethylamphetamine; 5-MeO-DMT, 5-methoxy-N,N-dimethyltryptamine; 5-MeO-MiPT, 5-methoxy-N-methyl-N-isopropyltryptamine; MDMA, 3,4-methylenedioxymethamphetamine; MDA, 3,4-methylenedioxyamphetamine; THCCOOH, 11-nor-9-carboxy-Δ9-tetrahydrocannabinol.

a Values represent the number of unique patients with at least one detected substance within the indicated drug category or class. Unless otherwise indicated, values represent analyte-specific positive detections. Because a patient could test positive for multiple substances, the summed analyte-specific counts may exceed the number of unique patients.

Logistic regression analysis was performed using TIDs as the reference category to estimate ORs for critical outcomes (ICU admission and ED death). Neither the NPS-positive nor the mixed NPS + TID–positive groups showed a statistically significant difference in the odds of developing critical outcomes compared with the TID group (Fig. 2A). Subgroup analysis was performed to further examine the association between individual NPS classes and severe outcomes. The forest plot illustrates that none of the NPS subclasses, including synthetic cannabinoids, cathinones, ketamine and its analogs, phenethylamines, tryptamines, designer benzodiazepines, or piperazines, were significantly associated with an increased risk of ICU admission or ED mortality compared with TID use. At 80% statistical power, the age- and sex-adjusted analysis could reliably detect odds ratios of approximately ≤0.36 or ≥2.20 for NPS versus TID exposure and ≤0.25 or ≥2.52 for mixed versus TID exposure. These findings indicate that the available sample size was primarily capable of detecting relatively large between-group associations, whereas smaller or moderate effects could have remained undetected.

**Fig. 2:**
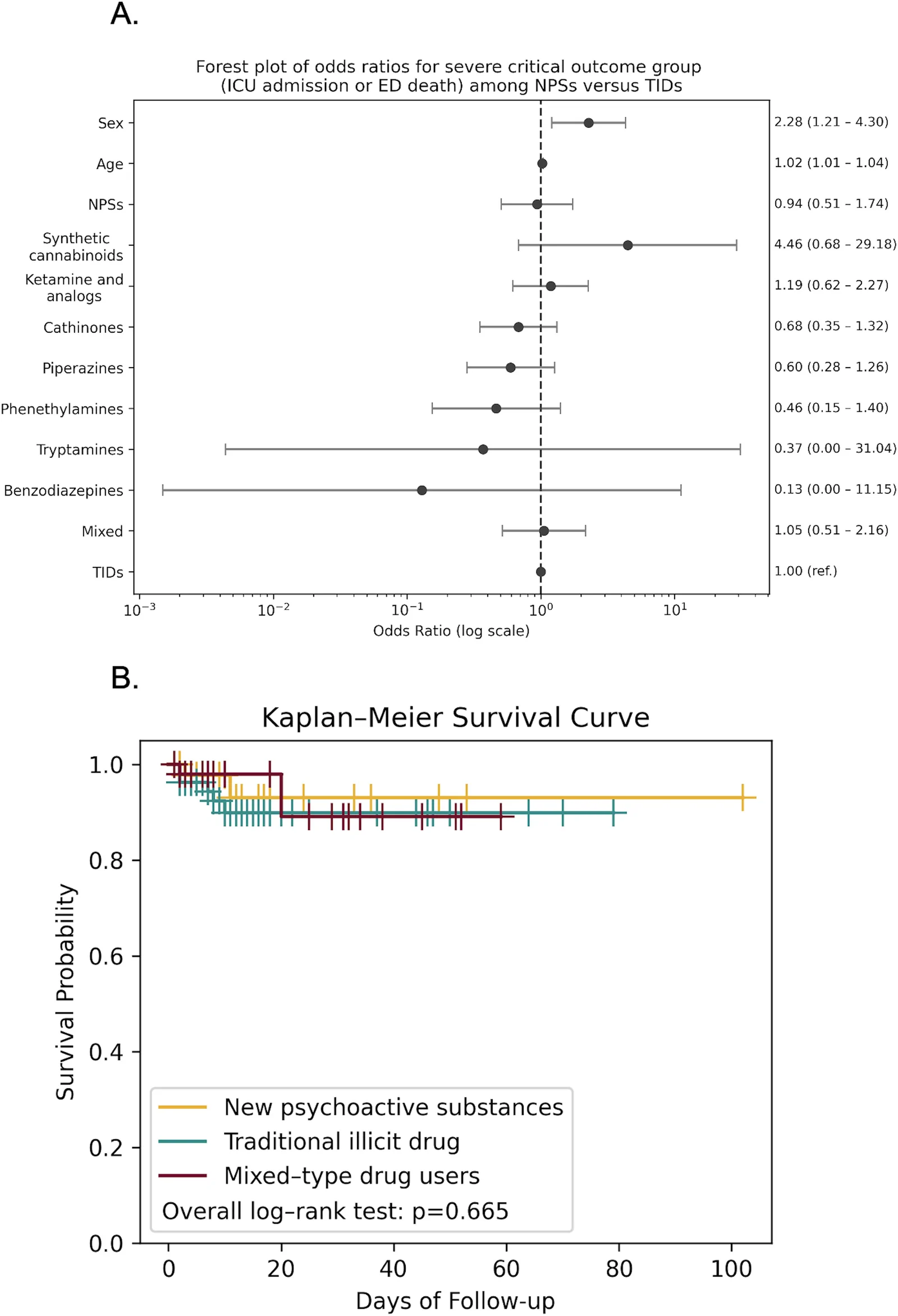
(A) Forest plot of odds ratios for critical outcomes (ICU admission or ED death) among patients taking new psychoactive substances (NPSs) and traditional illicit drugs (TIDs). Two multivariable logistic regression models were constructed using TIDs as the reference group. Model 1 (m₁) compared overall NPS, mixed, and TID users; Model 2 (m₂) compared NPS subgroups, mixed, and TID users. Both the models were adjusted for age and sex. (B) Kaplan–Meier survival curves comparing outcomes among the three groups of patients with positive urine toxicology results: new psychoactive substances (NPSs, yellow line), traditional illicit drugs (TIDs, teal line), and mixed-type users (maroon line). The curves represent survival probabilities during the hospital follow-up period. No statistically significant differences in survival were observed among the three groups (overall log-rank test, p = 0.665) (For interpretation of the references to colour in this figure legend, the reader is referred to the web version of this article).

In the ketamine reclassification sensitivity analysis, the NPS-only, TID-only, and mixed groups comprised 93, 157, and 112 patients, respectively. Critical outcomes occurred in 16.1%, 25.5%, and 16.1% of patients, respectively. The age- and sex-adjusted associations remained nonsignificant for NPS exposure (aOR, 0.75; 95% CI, 0.38–1.51) and mixed exposure (aOR, 0.82; 95% CI, 0.42–1.61), compared with TID exposure. Thus, reclassification of ketamine did not materially alter the primary interpretation. Similarly, additional adjustment for participating center yielded estimates consistent with the primary analysis (NPS vs. TID: aOR, 1.01; 95% CI, 0.54–1.91; mixed vs. TID: aOR, 1.06; 95% CI, 0.49–2.26), and the overall contribution of center was not statistically significant (likelihood-ratio test, p = 0.084). In sex-stratified analyses, the adjusted OR for NPS versus TID exposure was 1.01 (95% CI, 0.50–2.02) among male patients and 0.71 (95% CI, 0.18–2.74) among female patients. Although the point estimate was lower among female patients, the confidence interval was wide and included the null. Moreover, there was no evidence of effect modification by sex (p for interaction = 0.900), indicating that the association between exposure category and critical outcomes did not significantly differ between male and female patients.

A total of 15 patients died during hospitalization. Among these fatal cases, morphine (6/15, 40%) and methamphetamine (5/15, 33%) were the leading agents in the TID group, whereas synthetic cathinones (5/15, 33%) and ketamine analogs (3/15, 20%) were the most common in the NPS group. Because multiple substances could be detected in the same patient, these categories were not mutually exclusive. Kaplan–Meier survival analysis (Fig. 2B) demonstrated no significant differences in short-term survival among patients with different categories of detected substances (overall log-rank test, p = 0.665). These results suggest that survival outcomes did not significantly differ between NPS users and patients with traditional illicit drug poisoning.

In total, 698 pairwise co-detection events were identified among patients with multiple detected substances. The most frequent pairing occurred within the cathinone class itself (13.5%), indicating the frequent polyuse of structurally similar synthetic cathinones, such as eutylone, ephylone, dibutylone, and mephedrone. The next most common co-occurrences were cathinones combined with ketamine (9.7%) and methamphetamine (9.6%), highlighting a strong overlap between stimulant-type NPS and classic psychostimulants. Other frequent patterns included amphetamine–methamphetamine (6.6%), cathinones–MDMA (4.7%), and ketamine–methamphetamine (4.7%), suggesting overlapping exposure patterns that may reflect polysubstance use or mixed illicit drug preparations. Less frequent but noteworthy associations were found between phenethylamines and amphetamine/methamphetamine (≈3%), implying occasional co-use of hallucinogenic and stimulant-type NPS. Overall, these data indicate that the dominant mixed-use pattern in this cohort is stimulant–dissociative combinations, particularly among synthetic cathinones, amphetamine-type stimulants, and ketamine (Fig. 3).

**Fig. 3:**
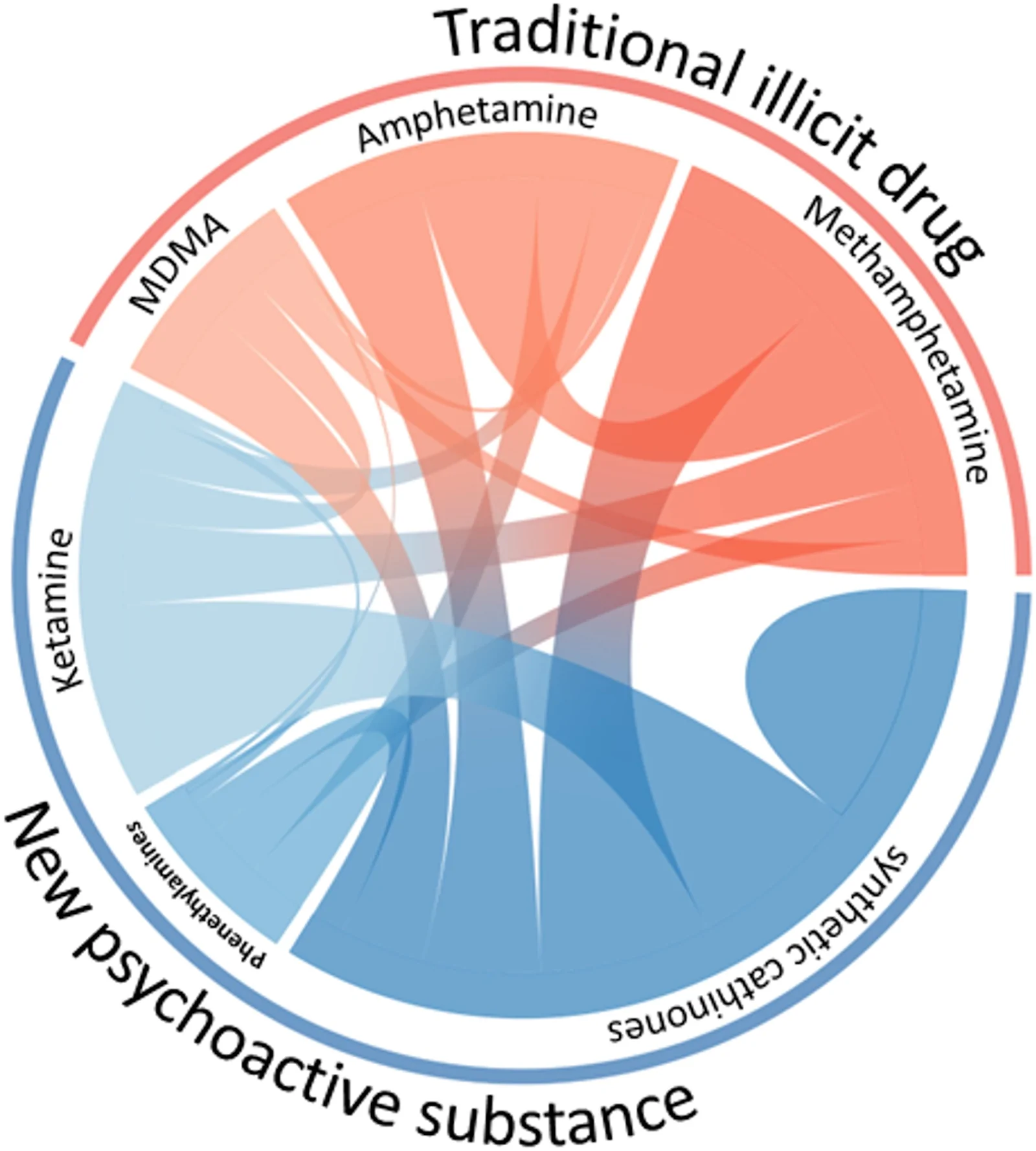
Patterns of codetection between new psychoactive substances (NPSs) and traditional illicit drugs. Chord diagram illustrating category-level codetection relationships among patients with multiple detected substances. For each patient, all unordered pairs of distinct detected substances were enumerated and subsequently grouped according to their corresponding drug categories; thus, a patient with *k* detected substances contributed *k*(*k*-1)/2 pairwise codetection events. Each arc represents a substance category, and each connecting ribbon represents a category-level codetection pair, with ribbon width proportional to the number of corresponding pairwise codetection events among the 698 total events. Within-category ribbons, such as cathinone–cathinone, represent concurrent detection of two distinct substances belonging to the same drug category. Blue segments denote NPSs (e.g., cathinones, phenethylamines, ketamine, and analogues), whereas red segments represent TIDs (e.g., methamphetamine, amphetamine, and MDMA). The most prominent connections were cathinone–cathinone, cathinone–ketamine, and cathinone–methamphetamine, illustrating that synthetic cathinones were frequently codetected with both ketamine and traditional psychostimulants (For interpretation of the references to colour in this figure legend, the reader is referred to the web version of this article).

## Discussion

In this multicenter cohort of analytically confirmed drug exposures, NPS exposure was not associated with significantly higher or lower odds of critical illness compared with TID exposure after adjustment for age and sex. Clinically important critical outcomes nevertheless occurred among patients with NPS exposure, and the overall interpretation remained qualitatively consistent in sensitivity analyses, including reclassification of ketamine as a traditional illicit drug. These findings indicate that NPS poisoning should not be assumed to represent a uniformly mild clinical entity. Emergency physicians should therefore remain vigilant and tailor monitoring and supportive care to the patient’s clinical presentation and severity. Although the confidence intervals were relatively wide, precluding a formal claim of equivalence between NPS and TID exposures, the present findings provide clinically relevant evidence regarding the severity spectrum of analytically confirmed NPS poisoning.

Before the widespread emergence of NPSs, surveillance data from North America before 2010 showed that acute poisoning rarely required intensive care, with only approximately 3% of patients admitted to critical care units [12]. Similarly, a nationwide French study (2009–2014) found that 4.2% of the patients required ICU admission or died in the hospital, with opioids and cocaine being responsible for the most severe outcomes [13]. Subsequently, a US ICU-based study reported that approximately 14% of hospitalized patients with poisoning required intensive care [14]. This is concordant with the 10% of NPS and TID poisoning cases requiring ICU care in Switzerland [6,9], and in Taiwan’s nationwide TEDAS project, 13.5% required ICU admission [15]. In our cohort, ICU admission occurred in 24 of 147 patients with NPS-only exposure (16.3%) and 29 of 130 patients with TID-only exposure (22.3%). The increasing proportion of critical outcomes among poisoned patients widely varies depending not only on the growing NPS abuse but also on the study design (prospective or retrospective), population (ED or ICU), and methods of toxicology testing (immunoassay or LC–MS/MS). Although these life-threatening cases are limited, they must be treated early and intensively, regardless of TID or NPS poisoning [14]. Notably, most critically ill patients achieved favorable outcomes with timely supportive management, which is consistent with a previous ICU toxicology study that demonstrated good recovery following early intervention [16].

In the present study, synthetic cathinones and ketamine were the principal agents associated with critical outcomes in the NPS group, whereas methamphetamine and morphine were the predominant agents in the TID group. The substance distribution observed among fatal cases differed from patterns reported in the United States and France, where opioids and cocaine accounted for the most life-threatening poisonings and deaths [13,17]. These differences may be explained by variations in illicit drug production regions, market prices, trafficking routes, and user preferences. Massive illicit fentanyl production in Mexico and China has led to its widespread adulteration with heroin, cocaine, and counterfeit pills, facilitating smuggling in the United States [18]. In contrast, Taiwan’s strict enforcement policies and geographic position have limited fentanyl influx, resulting in the predominance of methamphetamine, ketamine, and synthetic cathinones [15,19]. Furthermore, the US opioid epidemic has been fueled by liberal prescription practices and pain-management culture [20], whereas stimulant and club drug use in Taiwan is more closely associated with night-life and functional stimulant use [3,21].

A study from Switzerland reported that serious or potentially life-threatening manifestations were rare among patients presenting with NPS poisoning [9]. Several earlier studies have underestimated the frequency and severity of NPS-related toxicity because they relied primarily on self-reported data or included only a subset of analytically confirmed cases [[22], [23], [24]]. In contrast to these descriptive reports, our study first expanded the scope of analysis by incorporating objective clinical outcomes. We found that the risk of critical illness did not significantly differ between the NPS and TID users, emphasizing that NPS poisoning should not be considered less severe. This comprehensive analytical and clinical investigation provides new insights into the epidemiology and adverse effects associated with NPS use. Moreover, by integrating subgroup-specific analyses across the nine NPS categories, our study offers a more detailed understanding of NPS-related clinical severity and survival, contributing substantially to the literature.

Our chord diagram revealed that the most prominent linkages were centered on the methamphetamine/amphetamine–cathinone–ketamine triad, which represents a prototypical stimulant–dissociative combination. Clinically, such co-use often manifests as concurrent sympathomimetic overdrive, tachycardia, hypertension, and hyperthermia, along with dissociative-related airway compromise, agitation, and psychosis, resulting in heightened cardiovascular and neurological severity [25,26]. This constellation substantially increases the complexity of sedation, ventilation, and rhythm controlling strategies in the ICU. Stimulant–dissociative combinations may potentiate psychotic symptoms and behavioral dysregulation beyond single-agent intoxication [27,28]. From a public health perspective, these “mixed-type” consumption patterns highlight the need for routine toxicovigilance systems capable of detecting emerging street-level combinations and guiding early warning interventions. For intensivists, recognition of atypical presentations, such as mixed agitation–somnolence states or refractory hyperthermia, should prompt consideration of polysubstance-related mechanisms within the diagnostic and management plan.

This study has some limitations. First, as this was a retrospective chart review, potential information bias, selection bias and missing data could not be excluded. The context of substance use, including recreational use, chemsex, and the interval from substance use to ED presentation, was not systematically recorded and therefore could not be reliably evaluated. Second, urine samples were collected based on clinical suspicion, and the toxicology results were unavailable to emergency physicians at the time of decision-making. Thus, ICU admissions were purely determined by clinical severity, minimizing the selection bias against NPS-related cases. Third, the LC–MS/MS assay used a predefined analytical panel comprising approximately 100 target substances. Although the panel covered a broad range of NPSs and included selected designer benzodiazepines, it did not comprehensively assess conventional benzodiazepines, and GHB was not included. More recently emerging NPS classes, including semisynthetic cannabinoids, nitazenes, and orphine analogues, were also not covered by the analytical panel. In addition, blood alcohol concentration was measured selectively and was available in only 567 patients (41.1%). Therefore, concomitant exposure to central nervous system depressants may have been incompletely characterized, and residual confounding from these co-exposures cannot be excluded. Moreover, given the rapidly evolving NPS market, our findings should not be generalized to all currently circulating NPSs, particularly newer substance classes not represented in the analytical panel. Fourth, polysubstance use was frequent, limiting the ability to attribute outcomes to a single agent. Finally, the number of critical outcomes was limited, and the present study was therefore sufficiently powered primarily to detect relatively large associations. Smaller or moderate differences between NPS and TID exposures could have remained undetected; consequently, the absence of statistical significance should not be interpreted as evidence of equivalence. Larger multicenter studies with greater numbers of analytically confirmed exposures and critical outcomes are needed to obtain more precise effect estimates.

## Conclusion

No statistically significant difference in critical outcomes was detected between NPS and TID exposures. The increasing overlap between NPS and TID use highlights the urgent need for ongoing toxicological surveillance, expansion of LC–MS/MS analytical capacity, and enhanced clinician awareness to facilitate the timely recognition and management of emerging substance-related emergencies.

## Authors’ contributions

T-I Weng, H-Y Chen, C-C Lin, and C-C Fang conceptualized and designed the study. T-I Weng, H-Y Chen, Y-J Su, J-H Yu, S-W Liu, and Y-C Chen contributed reagents, materials, and analytical tools. P-H Liao, C-M Chang, and Y-C Chen performed the data analysis, developed the statistical models, and drafted the manuscript. T-I Weng, C-C Lin, and H-Y Chen interpreted the results. Yen-Chia Chen is the corresponding author and has the final responsibility for submitting the manuscript for publication. All the authors have read and approved the final version of the manuscript.

## Consent for publication

Not applicable.

## Ethics approval and consent to participate

The study protocol was reviewed and approved by the Institutional Review Boards (IRBs) of Taipei Veterans General Hospital, Taiwan (approval number: TVGH-2018-07-049BC); Chang Gung Medical Foundation, Taiwan (approval number: CGMH-202001418B0), MacKay Memorial Hospital, Taiwan (20MMHIS036e); National Taiwan University Hospital, Taiwan (201711102RIND); Hualien Tzu Chi Hospital, Taiwan (109-165-B); and China Medical University Hospital, Taiwan (CMUH110-REC3-038). The requirement for written informed consent was waived by the IRBs. All procedures were performed in accordance with the ethical standards of the Institutional and National Research Committees and the 1964 Declaration of Helsinki and its later amendments.

## Funding

This study received no specific funding.

## Availability of data and materials

The data collected for this study, including individual participant data and the data dictionary, will not be made publicly available because of IRB regulations and the sensitive nature of medical information.

## Declaration of competing interest

The authors declare no conflict of interest. The patients and the public were not involved in the research design, conduct, reporting, or dissemination plans.
