# Supplementary material for: The hidden severity of new psychoactive substances: clinical evidence from emergency and intensive care admissions in Taiwan
Source: Ann Intensive Care. 2026 Sep 28;16:100163. doi: 10.1016/j.aicoj.2026.100163 (PMC13677263; doi:10.1016/j.aicoj.2026.100163)
Supplement: Supplementary file 1 [file mmc1.docx]

Supplementary Table 1. Psychiatric, behavioral and clinical manifestations according to detected NPS class

|  | Synthetic cathinones  (N=107) | Ketamine analogues  (N=97) | Piperazines  (N=68) | Phenethylamines  (N= 32) | Designer benzodiazepines  (N=7) | Synthetic cannabinoids  (N=5) | Tryptamines  (N=2) |
| --- | --- | --- | --- | --- | --- | --- | --- |
| Altered mental status | 54 (50.47) | 44 (45.36) | 41 (60.29) | 20 (62.5) | 5 (71.43) | 4 (80.0) | 0 (0.0) |
| Bizarre behavior | 36 (33.64) | 23 (23.71) | 11 (16.18) | 14 (43.75) | 3 (42.86) | 1 (20.0) | 1 (50.0) |
| Suicide | 8 (7.48) | 12 (12.37) | 25 (36.76) | 4 (12.5) | 0 (0.0) | 2 (40.0) | 1 (50.0) |
| Self-harm | 13 (12.15) | 16 (16.49) | 16 (23.53) | 7 (21.88) | 1 (14.29) | 2 (40.0) | 0 (0.0) |
| Violence | 12 (11.21) | 8 (8.25) | 8 (11.76) | 5 (15.62) | 4 (57.14) | 0 (0.0) | 0 (0.0) |
| Delirium | 43 (40.19) | 28 (28.87) | 7 (10.29) | 15 (46.88) | 4 (57.14) | 2 (40.0) | 0 (0.0) |
| Hallucination | 12 (11.21) | 6 (6.19) | 3 (4.41) | 4 (12.5) | 1 (14.29) | 0 (0.0) | 0 (0.0) |
| First-time seizure | 6 (5.61) | 6 (6.19) | 0 (0.0) | 2 (6.25) | 0 (0.0) | 1 (20.0) | 0 (0.0) |
| Status epilepticus | 1 (0.93) | 1 (1.03) | 0 (0.0) | 0 (0.0) | 0 (0.0) | 1 (20.0) | 0 (0.0) |
| Myoclonus/rigidity | 5 (4.67) | 4 (4.12) | 3 (4.41) | 3 (9.38) | 0 (0.0) | 0 (0.0) | 0 (0.0) |
| Sympathomimetic symptoms | 12 (11.21) | 8 (8.25) | 4 (5.88) | 5 (15.62) | 0 (0.0) | 0 (0.0) | 0 (0.0) |
| Diaphoresis/sweating | 9 (8.41) | 6 (6.19) | 7 (10.29) | 4 (12.5) | 0 (0.0) | 0 (0.0) | 0 (0.0) |
| Headache | 6 (5.61) | 3 (3.09) | 2 (2.94) | 1 (3.12) | 1 (14.29) | 1 (20.0) | 0 (0.0) |
| Palpitation | 13 (12.15) | 8 (8.25) | 2 (2.94) | 1 (3.12) | 0 (0.0) | 0 (0.0) | 0 (0.0) |
| Facial flush | 5 (4.67) | 3 (3.09) | 6 (8.82) | 4 (12.5) | 0 (0.0) | 0 (0.0) | 0 (0.0) |
| Dyspnea | 12 (11.21) | 9 (9.28) | 5 (7.35) | 3 (9.38) | 0 (0.0) | 1 (20.0) | 0 (0.0) |
| Chest pain | 5 (4.67) | 7 (7.22) | 4 (5.88) | 0 (0.0) | 0 (0.0) | 0 (0.0) | 0 (0.0) |
| Nausea or vomiting | 8 (7.48) | 5 (5.15) | 3 (4.41) | 1 (3.12) | 1 (14.29) | 1 (20.0) | 0 (0.0) |
| Tremor | 4 (3.74) | 2 (2.06) | 3 (4.41) | 4 (12.5) | 0 (0.0) | 0 (0.0) | 0 (0.0) |
| Numbness | 1 (0.93) | 5 (5.15) | 2 (2.94) | 0 (0.0) | 0 (0.0) | 1 (20.0) | 0 (0.0) |
| Focal weakness | 0 (0.0) | 0 (0.0) | 1 (1.47) | 0 (0.0) | 0 (0.0) | 0 (0.0) | 0 (0.0) |
| Trauma case | 13 (12.15) | 13 (13.4) | 3 (4.41) | 1 (3.12) | 1 (14.29) | 0 (0.0) | 0 (0.0) |

Data are presented as n (%). Percentages were calculated using the number of patients with each detected NPS class as the denominator. NPS categories were not mutually exclusive; patients with multiple detected NPS classes could contribute to more than one column. Patients with concomitant TID exposure were included in this descriptive analysis.
